# Prediction of Acid-Base and Potassium Imbalances in Intensive Care Patients Using Machine Learning Techniques

**DOI:** 10.3390/diagnostics13061171

**Published:** 2023-03-18

**Authors:** Ratchakit Phetrittikun, Kerdkiat Suvirat, Kanakorn Horsiritham, Thammasin Ingviya, Sitthichok Chaichulee

**Affiliations:** 1 Department of Biomedical Sciences and Biomedical Engineering, Faculty of Medicine, Prince of Songkla University, Songkhla 90110, Thailand; 2 College of Digital Science, Prince of Songkla University, Songkhla 90110, Thailand; 3 Department of Family and Preventive Medicine, Faculty of Medicine, Prince of Songkla University, Songkhla 90110, Thailand; 4 Research Center for Medical Data Analytics, Faculty of Medicine, Prince of Songkla University, Songkhla 90110, Thailand

**Keywords:** critical care, machine learning, acid–base balance, prediction, big data, health informatics

## Abstract

Acid–base disorders occur when the body’s normal pH is out of balance. They can be caused by problems with kidney or respiratory function or by an excess of acids or bases that the body cannot properly eliminate. Acid–base and potassium imbalances are mechanistically linked because acid–base imbalances can alter the transport of potassium. Both acid–base and potassium imbalances are common in critically ill patients. This study investigated machine learning models for predicting the occurrence of acid–base and potassium imbalances in intensive care patients. We used an institutional dataset of 1089 patients with 87 variables, including vital signs, general appearance, and laboratory results. Gradient boosting (GB) was able to predict nine clinical conditions related to acid–base and potassium imbalances: mortality (AUROC = 0.9822), hypocapnia (AUROC = 0.7524), hypercapnia (AUROC = 0.8228), hypokalemia (AUROC = 0.9191), hyperkalemia (AUROC = 0.9565), respiratory acidosis (AUROC = 0.8125), respiratory alkalosis (AUROC = 0.7685), metabolic acidosis (AUROC = 0.8682), and metabolic alkalosis (AUROC = 0.8284). Some predictions remained relatively robust even when the prediction window was increased. Additionally, the decision-making process was made more interpretable and transparent through the use of SHAP analysis. Overall, the results suggest that machine learning could be a useful tool to gain insight into the condition of intensive care patients and assist in the management of acid–base and potassium imbalances.

## 1. Introduction

Patients in intensive care units (ICUs) usually suffer from severe or life-threatening diseases and injuries [1]. Patients are provided with multiple life support systems to maintain their physiological functions. They are at high risk of clinical deterioration, which can occur frequently, abruptly, and without warning. Such problems need to be identified early and treated immediately. They require attentive and specific care with state-of-the-art diagnostic and curative technologies to ensure their normal body function.

Acid–base balance refers to the balance of acidity and alkalinity in the human body [2]. Carbon dioxide (CO${}_{2}$) is crucial for acid–base balance in the body [3]. Patients may have a variety of conditions that can affect carbon dioxide balance, including respiratory disorders, renal dysfunction, and certain medications. Carbon dioxide balance is maintained by a delicate balance between the body’s production of carbon dioxide and its removal by the respiratory and renal systems. The respiratory system removes carbon dioxide through breathing, while the kidneys regulate carbon dioxide levels by controlling the excretion of bicarbonate through the urine. Proper carbon dioxide balance is important. Excess carbon dioxide in the blood (hypercapnia) can lead to acidosis. On the other hand, a lack of carbon dioxide in the blood (hypocapnia) can lead to alkalosis. In the ICU, carbon dioxide balance is carefully monitored to ensure that patients are receiving the appropriate level of ventilation and that their kidneys are functioning properly. If an imbalance is detected, medical intervention may be needed to correct it. This may include administering oxygen or mechanical ventilation to assist breathing, or administering medications to regulate carbon dioxide levels.

Acid–base status is a key factor in understanding the physiological changes occurring in critically ill patients, as well as in making diagnoses, developing treatment plans, and monitoring progress [4]. Acid–base imbalance is a deviation from the usual balance of acids and bases in the body that causes the plasma pH to deviate from its normal range [2]. The balance of acids and bases in the body is tightly regulated because even tiny deviations from the normal range can have serious consequences for numerous organs, some of which are life-threatening. The body regulates the acid–base balance of the blood through various processes, such as the lungs (change in respiratory rate), the kidneys (excretion of excess acid or base), and buffer systems (use of bicarbonate, ammonia, proteins, and phosphate) [5]. An excess of acid is called acidosis, while an excess of base is called alkalosis. The process causing the imbalance is classified according to the source of the disturbance (respiration or metabolism) and the direction of the pH change. This results in the four main processes: respiratory acidosis, respiratory alkalosis, metabolic acidosis, and metabolic alkalosis. Metabolic acidosis and metabolic alkalosis can occur due to an imbalance in the production and excretion of acids and bases by the kidneys, while respiratory acidosis and respiratory alkalosis are caused by changes in carbon dioxide exhalation due to lung or respiratory disease. Patients may experience multiple acid–base disturbances. To determine a patient’s acid–base balance, the physician needs to monitor the pH and the levels of carbon dioxide and bicarbonate in the blood. When an acid–base imbalance occurs, the body automatically attempts to compensate and restore the blood pH to normal through the respiratory and metabolic systems [2]. If the blood pH has changed significantly, this may indicate that the body’s ability to adapt is failing and further investigation and treatment of the underlying cause of the acid–base disorder is needed.

Over half of the body’s weight is made up of water. Electrolytes are minerals that carry an electric charge when they are dissolved in a liquid such as blood [6]. The kidneys help maintain electrolyte concentrations by filtering electrolytes and water from blood, returning some to the blood, and excreting any excess into the urine. Potassium is one of the electrolytes in the human body needed for the normal functioning of cells, neurons, and muscles. The body must maintain blood potassium levels within a certain range. High (hyperkalemia) or low (hypokalemia) levels of potassium in the blood can have disastrous consequences, such as cardiac arrhythmias or even cardiac arrest. The body can use the large reserves of potassium in the cells to keep blood potassium levels constant. Healthy kidneys can adjust potassium excretion to match fluctuations in potassium intake. Some medications and diseases can interfere with the transport of potassium, significantly affecting blood potassium levels. The interaction between acid–base and potassium balance involves transcellular cation exchange as well as changes in kidney function [7]. An imbalance in acid–base balance can lead to shifts in the transport of potassium into and out of cells [5]. This is particularly evident in metabolic acidosis, metabolic alkalosis, and, to a lesser extent, respiratory acid–base disorders. Hyperkalemia and hypokalemia can be detected during a routine blood test or when a physician detects certain abnormalities in an electrocardiogram. Physicians may assess the amount of potassium excreted in the urine or look for signs of diabetes, acidosis, or kidney disorders.

Generally, patients admitted to the ICU are evaluated based on clinical, pathological, and physiological data. They may experience worsening of symptoms or complications due to conditions such as heart failure. Often, the patient’s worsening condition and dysfunction is not directly indicated, resulting in a delay in assessing the patient’s risk and response to changes in the patient’s condition. This can lead to more severe disease and loss of life. Timely treatment is therefore essential. Therefore, early detection can help patients have a better chance of survival. It can also reduce the use of medical resources.

Early detection of critical clinical conditions can lead to better health outcomes and lower healthcare costs [8]. Early warning scores (EWS) have been used to assess and determine a patient’s severity based on clinical parameters routinely collected during an ICU stay [9]. Examples include Acute Physiology and Chronic Health Evaluation (APACHE) [10], Simplified Acute Physiology Score (SAPS) [11], Modified Early Warning Score (MEWS) [9], and National Early Warning Score (NEWS) [12]. These EWS scores employ pathological data, physiological data, and patient responses for calculation. They alert clinical staff when a patient’s severity falls into the abnormality range, prompting them to immediately attend to the patient and plan treatment. However, most assessment tools were originally developed for manual bedside calculation. As electronic health records (EHRs) become ubiquitous, these tools are now closely integrated with modern EHRs. This allows scores to be calculated automatically based on patient data in EHRs. This makes patient care more convenient, faster, and more timely.

Machine learning has been shown to be able to find relationships in medical data that change over time with different patient conditions [13]. With patient data available in EHRs and real-time vital signs at the bedside, such as patient personal data (gender, age, and underlying disease), treatment history, physiological data, and laboratory results, it could be possible to develop an algorithm to determine the relationship between dynamic medical data and critical patient conditions by examining the relationships that occur around the patient at a critical time [14]. This could lead to modern data-driven EWS development that could be more accurate, specific, and real-time. It could also help physicians make informed decisions, making the process of patient care safer.

Many studies have shown that machine learning can predict or classify the clinical condition and clinical outcomes in intensive care patients [15,16,17,18,19,20,21,22,23,24,25,26,27,28,29]. For early prediction of sepsis, Kam et al. [15] developed a long short-term memory (LSTM) model, a deep learning model that incorporates past information, for early detection of systemic inflammatory response syndrome (SIRS) conditions that could lead to sepsis. Nemati et al. [16] developed a modified Weibull–Cox proportional hazards model for sepsis detection using data from EHR and high-resolution bedside monitoring. Zhang et al. [17] developed an LSTM model to predict sepsis using data (demographics, vital signs, laboratory values, and nutrition) from over 10,000 individual patients. Although these studies examined similar outcomes, the results cannot be compared because they used different datasets, and the definition of the outcomes was also different. Several studies investigated other critical clinical conditions. Kwon et al. [18] developed a deep learning algorithm for in-hospital cardiac arrest prediction. Tomasev et al. [19] developed a recurrent neural network model for continuous prediction of acute kidney injury. Wanyan et al. [20] developed a recurrent neural network model with contrastive loss to predict mortality, intubation, and ICU transfer in hospitalized COVID-19 patients. More recently, some studies employed more comprehensive clinical data and examined less critical clinical events. Lee et al. [21] used an autoregressive event time series model to predict the future occurrence of clinical events defined as drug administration, laboratory orders, medical procedures, and physiological measurements. Their model consists of three mechanisms with LSTM to process information from the distant past, a linear transformation model module to process recent information, and a probabilistic model to process periodicity. Their model can handle complex multivariate temporal time series of ICU data. Kaji et al. [22] trained LSTM to predict sepsis, myocardial infarction, and vancomycin antibiotic administration. Recently, some studies have begun to examine specific clinical conditions, such as hypocapnia [25], hypokalemia [27], hyperkalemia [28], and acid–base disturbances [29], but did not utilize time-series data. Many of the studies showed promising results with high performance measures, which can be further investigated in the clinic.

Although prediction by machine learning has the chance to improve the patient’s health status, the problem with the reliability of the predictions is that they are not reliable for physicians because they are not interpretable. The problem can be addressed by applying an explanatory tool to the model so that it can provide the meaning of each predicted parameter (such as pulse, respiratory rate, and creatinine) for the prediction of critical illness (such as sepsis, acute kidney injury, acute lung injury). Current research trends address the use of interpretable machine learning models that can incorporate comprehensive and past information for early prediction of important clinical conditions, which could lead to early intervention, which in turn could lead to better patient outcomes.

Currently, machine learning development in the ICU is largely focused on predicting key outcomes, such as mortality, length of stay, and sepsis [15,16,17,22,23,24]. In other areas, such as acid–base disturbances and potassium imbalances, there are significant gaps that remain to be explored [25,26,27,28,29]. The acid–base and electrolyte balance is essential for the optimal functioning of physiological processes and cells, and an imbalance is often the result of an underlying disease and can have negative effects on clinical outcomes. Determining and, if possible, predicting the acid–base status of patients and using this information to control or regulate the balance can be beneficial in managing underlying diseases. The aim of this study is to investigate machine learning models to predict the occurrence of acid–base and potassium imbalances in intensive care patients. We used comprehensive patient data from Songklanagarind Hospital in Thailand. We employed 87 clinical predictors, including vital signs, general patient appearance, and laboratory measurements (chemistry labs, hematology labs, microscopy labs, and arterial blood gases).

## 2. Materials and Methods

### 2.1. Dataset

This study involved the de-identified data extracted from the EHR of Songklanagarind Hospital in Thailand. We used the vital signs, general appearance, and laboratory results of patients admitted to the hospital from August 2019 to April 2022 who spent at least 24 h in the medical intensive care unit (MICU). Our laboratory results involved blood chemistry tests, hematology tests, microbiology tests, and arterial blood gases. We included only the first MICU visit and excluded subsequent visits. We excluded patients in whom the duration of recorded vital signs and laboratory tests was less than 24 h and patients in whom all four laboratory tests were not examined during their ICU stay. Our dataset included 1089 patients with 1137 hospital admissions. Table 1 shows the characteristics of the patients in our dataset. Our study was approved by the Office of Human Research Ethics Committee, Faculty of Medicine, Prince of Songkla University (REC. 63-541-25-2).

### 2.2. Clinical Variables

**Figure 1:**
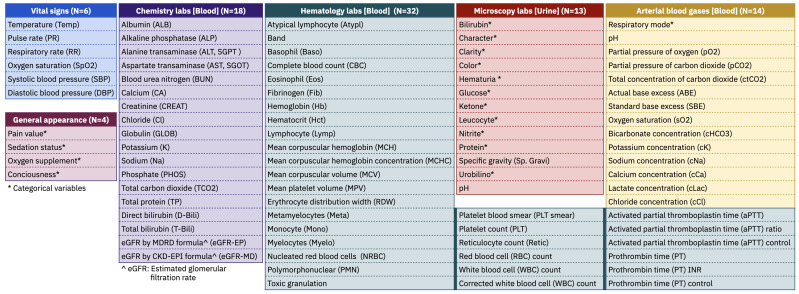
Clinical variables in our dataset.

In this study, we used vital signs, general appearance of the patient, and laboratory measurements as predictors. Vital signs and general appearance represent the important body functions of the patient. They are frequently monitored and carefully recorded in the EHR. Laboratory results are often used to predict the patient’s current clinical condition. We considered blood chemistry tests, hematology tests, microbiology tests, and blood gas tests. We selected only those variables for which there was an average of at least one measurement per day, calculated from the days patients spent in the MICU, for all patients. This resulted in a total of 87 variables. Figure 1 contains a list of all clinical variables considered in our study.

### 2.3. Clinical Conditions

Our study aims to predict clinical conditions that are common in intensive care patients so that early intervention can help improve patient outcomes. We identified 9 clinical conditions that occur in patients in our dataset: mortality, hypocapnia, hypercapnia, hypokalemia, hyperkalemia, metabolic acidosis, metabolic alkalosis, respiratory acidosis, and respiratory alkalosis. The criteria for hypocapnia and hypercapnia were defined according to Laserna et al. [30]. For hypokalemia and hyperkalemia, the European Resuscitation Council Guidelines for Resuscitation 2010 were used [31]. Regarding acid–base balance disorders, our study used the physiological approach according to Berend et al. [32] and Constable et al. [33]. Table 2 shows the criteria of each clinical condition.

#### 2.3.1. Mortality

Mortality is defined as the patient’s death while in the ICU. Mortality is a common prediction target and can serve as a benchmark for the algorithm. It is strongly associated with clinical variables in the EHR.

#### 2.3.2. Hypocapnia and Hypercapnia

Hypocapnia is present when a pCO_2_ level is less than 35 mmHg, while hypercapnia is present when a pCO_2_ level is more than 45 mmHg [30]. They may lead to an acid–base imbalance. Low carbon dioxide levels lead to a decrease in the hydrogen ion concentration in the blood, making the pH more basic. On the other hand, high carbon dioxide levels lead to an increase in the hydrogen ion concentration in the blood, making the pH more acidic. Patients may complain of lethargy, mild headache, shortness of breath, nausea, hyperventilation or hypoventilation, or fatigue [34].

#### 2.3.3. Hypokalemia and Hyperkalemia

More than 20 % of hospitalized patients were found to have hypokalemia, i.e., a K^+^ of less than 3.5 mmol/L [31]. With lower serum potassium levels, there is a risk of muscle necrosis, which can develop into paralysis, with deterioration of respiratory function and an increase in cardiac arrhythmias [35].

Hyperkalemia can be life-threatening, especially in patients with chronic kidney disease (CKD), diabetes mellitus, or heart failure. Hyperkalemia is often caused by stress, illness, or dehydration. A K^+^ of greater than 5.5 mmol/L is recommended as a threshold for treatment of hyperkalemia [31].

#### 2.3.4. Metabolic Acidosis and Metabolic Alkalosis

Metabolic acidosis is present when there is a cHCO_3_^−^ of <22 mmol/L and a pH of <7.35 [32,33]. Patients with metabolic acidosis can have serious consequences for cellular function and an increased risk of disease and death.

Compensatory hypoventilation in critically ill patients may lead to hypoxia or pulmonary infection. Failure of the right compensatory ventilation results in an increase in pCO_2_ that precipitates metabolic alkalosis, the criteria for which are a cHCO_3_^−^ of >26 mmol/L and a pH of >7.45 [36].

#### 2.3.5. Respiratory Acidosis and Respiratory Alkalosis

Respiratory acidosis often occurs when the lungs are unable to remove all carbon dioxide produced by the body. It affects approximately 25% of patients with chronic respiratory failure. Patients with respiratory acidosis have a pH of <7.35 and a pCO_2_ of >45 mmHg [32,33]. Respiratory acidosis is associated with a higher risk of mortality and a greater need for intubation [37].

Respiratory alkalosis is often caused by hyperventilation which most commonly occurs in response to hypoxia, metabolic acidosis, increased metabolic demands, and pain. The criteria for respiratory alkalosis are a pCO_2_ of <35 mmHg and a pH of >7.45 [32,33].

#### 2.3.6. Annotation of Clinical Conditions

We used laboratory measurements, i.e., blood chemistry and arterial blood gas values, to identify clinical conditions. We considered only the first occurrence of the same clinical condition for each admission. All clinical conditions were annotated using variable values in the EHR data without physician involvement. We considered the time of occurrence of a clinical condition to be the time when the associated laboratory values met the criteria. Table 2 shows the number of admissions with presence of each clinical condition identified in our dataset.

### 2.4. Data Preparation

**Figure 2:**
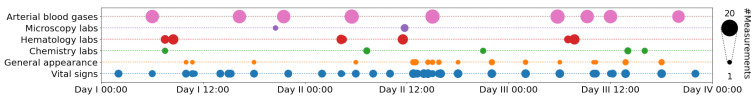
Illustration of our sequential clinical data randomly selected from a patient in the dataset during their 72 h stay in the ICU. The graph shows how often the clinical variables were measured, with more measurements during the day than at night.

This study used the vital signs and laboratory results around patients admitted into the ICU. These data were mixed between numerical and text values. We converted the numerical values of the same variable into the same scale. We encoded all discrete and text values into categories. For each patient and variable, a time series with a fixed 15 min interval was created and filled with measurements taken during the associated time interval. The 15 min interval was chosen as our sampling period based on the observation that vital signs and general appearance factors can be collected as frequently as every 15 min. If we had chosen a larger interval, our machine learning models may have lost the ability to learn about the temporal dynamics of clinical data. If we had chosen a smaller interval, the number of observations would have been too high. Missing values were filled using the fill-forward method, from the last observation to the next values, taking into account all vital signs and laboratory measurements obtained before admission to the MICU but during the same hospital stay. This was performed to avoid sparse time series that could lead to poor results. Figure 2 shows examples of time series for one patient, which illustrate the fluctuating character of data in intensive care patients.

### 2.5. Machine Learning Models

Our problem was cast as binary classification. We used a 12 h observation window. This was not a problem in patients who had recently been transferred to the ICU because the values of the clinical variables had been carried forward since the beginning of their stay when they were in other units. We investigated the performance of machine learning algorithms for predicting each clinical condition $T_{P}=\left\{1,2,4\right\}$ hours before its occurrence.

**Figure 3:**
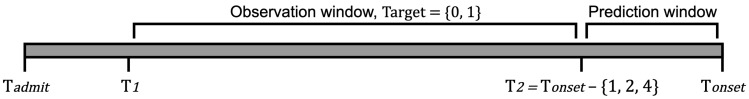
Diagram of our predictive tasks. The data in the observation window between $T_{1}$ and $T_{2}$ were used to predict the clinical condition occurring at time $T_{onset}$. For the positive sequences, the time of onset corresponded to the time of occurrence of the clinical condition. For the negative sequences, the time of onset was randomly chosen within the admission.

For each clinical condition, patients in whom the clinical condition occurred during their stay in the ICU were assigned to the positive group, whereas patients in whom the clinical condition did not occur were assigned to the negative group. For the positive group, we extracted the clinical signals in a 12 h observation window $T_{P}$ hours before the onset of the clinical condition. For the negative groups, we extracted a 12 h observation window from the clinical signals at random for each patient (see Figure 3).

Regarding the evaluation procedures, our dataset included 1089 patients with 1137 admissions. A model received time-varying vital signs and laboratory results as inputs and generated the probability risk for each clinical condition between 0 and 1. We addressed data imbalance by performing sample weighting during the training of an algorithm.

We investigated four discriminative algorithms: *K* Nearest Neighbours (KNN), Support Vector Machine (SVM), Random Forest (RF), and Gradient Boosting (GB). Our goal was to explore simple but powerful classifiers that can be implemented at the edges. We conducted all experiments using the Python programming language and utilized SQL for retrieving all data from the database. We used the Scikit-learn (v.1.1.1), SHAP (v.0.41.0), Pandas (v.1.4.2), Numpy (v.1.21.6), and Matplotlib (v.3.5.2) frameworks.

#### 2.5.1. K Nearest Neighbours

KNN is a versatile algorithm that works by finding the *K* closest data points to a given query point and uses the majority class of these data points as a prediction for the query point. With regards to hyperparameter optimization, we examined different numbers of neighbors ({3, 5, 7, 9}) and different weight functions (uniform and distance weights).

#### 2.5.2. Random Forests

RF is a collection of decision trees, where each tree is trained on a randomly selected subset of the training data. The predictions of each tree are then combined to make a final prediction by considering the majority of votes. Random Forest can reduce overfitting, which is a common problem with decision trees, by training each tree on a different subset of the training data. RF can handle high-dimensional large data because it is inherently parallel. The weight associated with each leaf node reflects the importance of the variables. With regards to hyperparameter optimization, we examineddifferent numbers of trees in the forest (200, 400, 600) and maximum depth of the trees ({4, 8, 12, 16}).

#### 2.5.3. Support Vector Machine

SVM is an algorithm that aims to find the hyperplane that maximally separates the different classes. SVM uses the kernel trick to transform the data into a higher dimensional space. The algorithm can effectively handle complex relationships in high-dimensional spaces between the features and the target with with high accuracy and reproducibility. For hyperparameter optimization, we examined different kernel types of trees in the forest (polynomial and radial basis function) and different regularization parameters (C parameter) ({1, 10, 100, 1000}).

#### 2.5.4. Gradient Boosting

GB is an ensemble method in which a sequence of weak learners is trained, with each successive learner trained to correct the errors of the previous learner. The final prediction is produced by combining the predictions of all individual learners. GB is able to handle missing values, outliers, and a large number of features and is resistant to overfitting. Unlike SVM, GB can automatically learn nonlinear complex relationships in the data without explicit mapping. GB has achieved state-of-the-art results on many machine learning tasks. For hyperparameter optimization, we examined different numbers of boosting stages ({100, 200, 400}) and maximum depth of the tree ({4, 8, 12}).

### 2.6. Evaluation Metrics

A true positive (TP) is when a model correctly identifies a positive class; a true negative (TN) is when a model correctly identifies a negative class; a false positive (FP) is when the model incorrectly identifies a negative class, and a false negative (FN) is when the model incorrectly identifies a positive class. Sensitivity or recall represents the proportion of actual positives that are correctly predicted:

(1) $$\mathrm{Sensitivity}\mathrm{or}\mathrm{Recall}=\frac{\mathrm{TP}}{\mathrm{TP}\;+\;\mathrm{FN}}.\;$$

Specificity represents the proportion of actual negatives that are correctly predicted:

(2) $$\mathrm{Specificity}=\frac{\mathrm{TN}}{\mathrm{TN}\;+\;\mathrm{FP}}.\;$$

Precision represents the proportion of positive predictions that are actually correct:

(3) $$\mathrm{Precision}=\frac{\mathrm{TP}}{\mathrm{TP}\;+\;\mathrm{FP}}.\;$$

F1 score is a harmonic mean of precision and recall and represents a single metric that indicates how well a model finds relevant results:

(4) $$F_{1}\mathrm{Score}=\frac{2\mathrm{TP}}{2\mathrm{TP}\;+\;\mathrm{FP}\;+\;\mathrm{FN}}.\;$$

A receiver operating characteristic (ROC) curve is a graphical representation that shows the trade-off between sensitivity and specificity by varying the classification threshold. A precision recall (PR) curve is similar to an ROC curve, but it shows the trade-off between precision and recall by varying the classification threshold. The PR curve is useful for evaluating the performance of a classifier when the class distribution is imbalanced (meaning that one class is significantly more prevalent than the other). Both the ROC curve and the PR curve are commonly used to evaluate the performance of binary classifiers. The area under the ROC curve (AUROC) and the area under the PR curve (AUPRC) are common metrics that can be used to evaluate the overall performance of a model.

Our study considers not only predictions but also understandable explanations. We used Shapley values [38] to determine how much each of the clinical features contributed to the prediction.

## 3. Results

The present study utilized clinical data from 1089 patients, encompassing 1137 admissions to the intensive care unit (ICU). A total of 87 clinical variables were considered, including vital signs, general appearance, chemical laboratories, hematology laboratories, microscopic labs, and arterial blood gases. The objective of the study was to predict nine clinical conditions: mortality, hypocapnia, hypercapnia, hypokalemia, hyperkalemia, metabolic acidosis, metabolic alkalosis, respiratory acidosis, and respiratory alkalosis. Although we used time series with a fixed 15 min interval as input, predictions can be made as the data come in, but the data must be formatted in time series with a fixed 15 min interval.

The results of the classification algorithms in predicting clinical conditions on the test set are presented in Table 3. The evaluation was conducted using a 12 h observation window and a 1 h prediction window, and the aim was to predict whether a clinical condition would occur within the subsequent hour. Precision, sensitivity (recall), specificity, and F1 score were calculated using the threshold that yielded the highest F1 score in the validation set.

**Figure 4:**
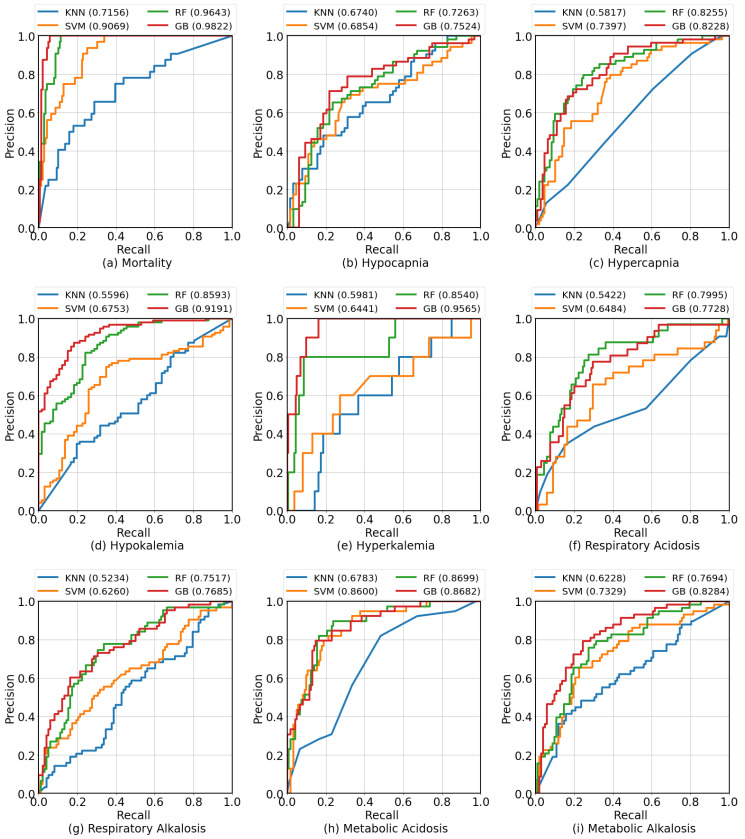
The ROC curves compare different algorithms for each target clinical condition. GB outperformed the other algorithms in most cases. There was a small number of positive cases for hyperkalemia.

We present both AUROC and AUPRC because the number of positive cases for each clinical condition varied. The former metric, AUROC, aims to minimize false negatives, while the latter metric, AUPRC, aims to minimize false positives. Based on the results presented in Table 3, the GB algorithm performed better than the other algorithms in 7 out of 9 clinical conditions: mortality (AUROC = 0.9822), hypocapnia (AUROC = 0.7524), hypokalemia (AUROC = 0.9191), hyperkalemia (AUROC = 0.9565), respiratory acidosis (AUROC = 0.8125), respiratory alkalosis (AUROC = 0.7685), and metabolic alkalosis (AUROC = 0.8284). The RF algorithm slightly outperformed GB in the remaining two clinical conditions: hypercapnia (AUROC = 0.8228) and metabolic acidosis (AUROC = 0.8682). The KNN algorithm was not effective in predicting any of the clinical conditions. The SVM algorithm performed competitively, but its performance did not surpass those of GB and RF. The clinical conditions that demonstrated the highest scores were mortality (AUROC = 0.9822 and AUPRC = 0.8557) and hypokalemia (AUROC = 0.9191 and AUPRC = 0.9455). In terms of predicting acid–base imbalances, the performance of the algorithms was similar, with AUROCs ranging between 0.7685 and 0.8699 and AUPRCs ranging between 0.5945 and 0.7150. Figure 4 shows the ROC curves compared for different classification algorithms for each clinical condition. Figure S1 shows the graphical comparison of F1 Score, AUROC, and AUPRC across different algorithms for each clinical condition.

Both precision and recall are important measurements because they provide different insights into the performance of a model. In some applications, a high recall rate may be more crucial in order to minimize the number of false-negative results, such as in screening tests. Conversely, a high precision rate may be more critical in applications where the cost of false-positive results is high, such as in diagnostic tests. The F1 score is a single metric that provides a balance between precision and recall and is less affected by imbalanced data. We used the decision threshold that gives the highest F1 score in the training set as the threshold for the test set to obtain precision, sensitivity, specificity, and F1 score. The clinical condition with the highest F1 score is hypokalemia (F1 = 0.8691), followed by mortality (F1 = 0.8101) and hypocapnia (F1 = 0.7115), respectively. Our F1 scores are in line with their AUROCs and AUPRC scores.

Table 4 shows the predictive performance of GB at different prediction windows (T${}_{P}$ = {1,2,4,8}) on the test set. The performance of the prediction algorithm decreased as the gap between the prediction and the onset increased. This was expected and due to the fact that the further into the future the prediction was made, the more uncertainty there was about the outcome. Figure S2 shows the graphical comparison of F1 Score, AUROC, and AUPRC across different early prediction periods for each clinical condition.

**Figure 5:**
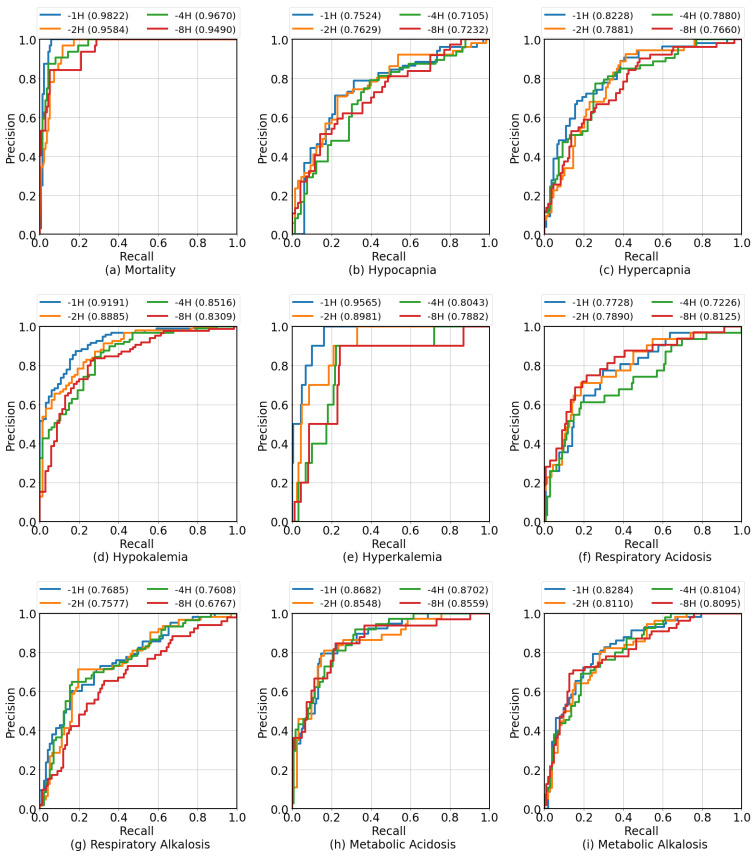
The ROC curves compare different early prediction periods (1, 2, 4, and 8 h before onset) using GB for each target clinical condition.

Figure 5 presents ROC curves at different early prediction periods (T${}_{P}$ = {1,2,4,8} h before onset). These ROC curves illustrate how the performance of the GB classifier changes as the prediction window increases. It appears that some clinical conditions were more robust towards early prediction than others as the performance of the classifier was relatively stable as the prediction window increased. Based on the results presented in Table 4, small decreases in AUROC and AUPRC (less than 0.05) were observed in the clinical conditions of mortality, hypocapnia, metabolic acidosis, and metabolic alkalosis when the prediction window was extended from 1 to 8 h. However, large decreases were observed in the clinical conditions of hypercapnia, hypokalemia, hyperkalemia, respiratory acidosis, and respiratory alkalosis.

**Figure 6:**
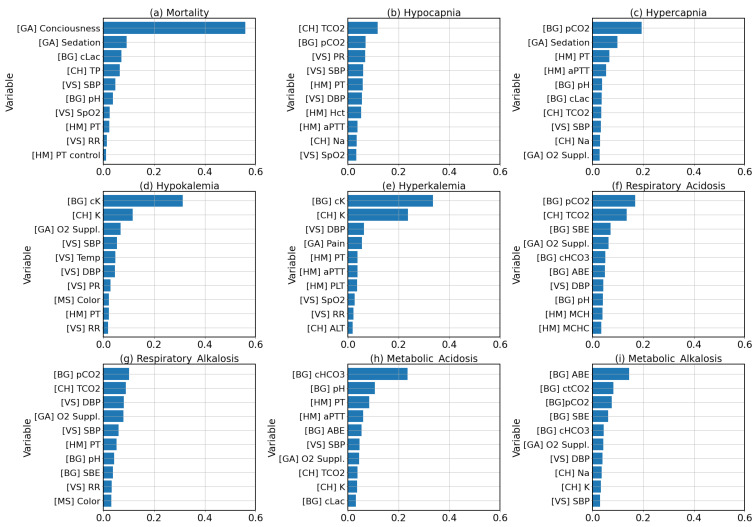
Feature importances calculated from the GB classifer for each clinical condition.

Figure 6 shows the importance of each clinical variable on the GB classifier on each clinical condition. The feature importance was calculated using the impurity-based Gini importance. This method quantifies the importance of each feature by measuring the decrease in node impurity that results from splitting on that feature. The total decrease in node impurity was normalized by the proportion of samples reaching that node and was averaged across all trees in the ensemble model. The resulting value reflect the influence of that feature on the model’s predictions.

According to Figure 6, patient consciousness was a significant factor in predicting mortality. The features related to carbon dioxide in the blood would be ranked highly for predicting hypocapnia and hypercapnia, as these clinical conditions are characterized by abnormal levels of carbon dioxide in the blood. Similarly, the features related to potassium would be ranked highly for predicting hypokalemia and hyperkalemia, as these clinical conditions are characterized by abnormal levels of potassium in the body. For acid–base imbalances, features related to acid, base, bicarbonate, and carbon dioxide would be ranked highly, as these factors play a key role in regulating acid–base balance in the body.

## 4. Discussion

Machine learning can be a tool to provide timely decision support, simplify the vast amount of information commonly available in the ICU, and highlight the most important elements for each patient. This study focused on the prediction of acid–base and potassium imbalances in intensive care patients. We also looked into predicting mortality, which was used as a baseline to demonstrate the effectiveness of our data embedding strategy and the prediction algorithm in a typical scenario. Our mortality prediction results were in line with other studies [20,23,24] despite ours employing our institutional dataset. Table 3 and Figure 4 indicate that tree-based algorithms, i.e., RF and GB, outperformed the other algorithms forthe early prediction of one hour. In general, all of the algorithms used in the study performed better than a random guess. Based on the results, mortality and hypokalemia were the two clinical conditions with the highest F1 Scores, AUROCs, and AUPRCs. Hypocapnia, hypercapnia, respiratory alkalosis, and metabolic alkalosis all had AUROCs of more than 0.65, indicating that they have potential for further study as clinical conditions that can be accurately predicted using machine learning.

When optimizing the hyperparameters, we observed that for KNN, a larger number of neighbors tended to lead to better performance, as this increases the probability that similar data points are present in the neighborhood. For SVM, the regularization parameter C controls the trade-off between maximizing the margin and minimizing the misclassification error. Larger values of C tended to lead to better results, as an attempt was made to increase the margin while attempting to correctly classify all training points. For RF, a larger number of trees tended to lead to better performance because the probability of overfitting was reduced, while the criteria used for data partitioning tended not to have a large effect on performance. For GB, the number of decision trees and the maximum depth of each decision tree were important parameters. We found that a larger number of trees and a lower maximum depth tended to lead to better performance. We observed slightly different sets of hyperparameters for each clinical condition for each model.

With regards to algorithms, GB and RF differ in how the trees are built and how their predictions are combined. GB builds a sequence of weak learners. GB adjusts the weights of data points misclassified by the previous tree to give more weight to difficult cases. RF independently trains many fully grown decision trees and then votes on classifying new data. RF is generally good when there are many features or when the data are high-dimensional. RF can effectively decorrelate the trees and reduce overfitting. RF also handles missing data and categorical variables well. GB is more prone to overfitting than RF, but it can find nonlinear interactions between variables. GB is also sensitive to the scale of the feature; few features may dominate the model. Both RF and GB can work well in different situations. and the choice depends on the dataset and specific problem. For hypercapnia, respiratory acidosis, and metabolic acidosis, it happens that RF scored higher than GB. We suspect that GB may have difficulty handling the complexity and may overfit in these problems.

From the results in Table 4 and Figure 5, the scores for respiratory acidosis and respiratory alkalosis decreased significantly as the prediction window increased. The scores for mortality, hypokalemia, and metabolic alkalosis remained relatively robust, even as the prediction window increased, allowing predictions for these clinical conditions to be made several hours in advance. The scores for hypokalemia decreased as the prediction window increased, eventually making early prediction more difficult. This may be due to the small number of positive samples for hyperkalemia. It is generally more difficult to make accurate predictions with a small number of positive samples, as there is less data available to train the prediction algorithm. This suggests that, due to their intrinsic characteristics, certain clinical conditions may be more difficult to predict than others, even with the use of machine learning techniques. With regards to the four cardinal acid–base disorders, the results suggest that the machine learning models seemed to learn about the physiological regulation of the HCO_3_^−^/CO_2_ buffering system and were able to predict the acid–base disorders. These models may be effective in providing decision support for nurses and clinicians in the ICU setting.

### 4.1. Comparison to Other Studies

Many studies have demonstrated the ability of machine learning to predict clinical outcomes and clinical conditions in intensive care patients. The performance of a model may vary depending on the specific dataset and prediction task. Most studies have been conducted in a domain-specific context, such as predicting clinical events that may occur during surgery. For hypocapnia, Chen et al. [25] developed machine learning models to predict six different outcomes, including hypocapnia, using GB with physiological signals. The authors obtained an AUROC of 0.8551 and an AUPRC of 0.4451. Their results with GB outperformed those of the LSTM. Our study achieved AUPRCs of 0.6099–0.6442, which were higher than those of the authors. For hypercapnia, Fan et al. [26] developed an RF model to predict hypercapnia during one-lung ventilation using RF. They obtained an AUROC of 0.7450, which is comparable to our study, which obtained AUROCs of 0.7660–0.8228. Regarding hypokalemia and hyperkalemia, Zhou et al. [27] developed a GB model for predicting severe hypokalemia with an AUROC of 0.73. Our study obtained higher AUROCs of 0.8309–0.9191 for hypokalemia. Similarly, Kwak et al. [28] investigated the prediction of hyperkalemia with an AUROC of 0.85 for both RF and GB. Ours achieved AUROCs of 0.7882–0.9565. For the four acid–base disorders, Cherif et al. [29] developed a mathematical model for predicting acid–base disorders that takes into account the physiological regulation of the buffer system. The model can predict the primary disturbances and provides pathophysiological insights. This study supports our hypothesis that the acid–base disorders can be predicted using machine learning algorithms.

### 4.2. Calibration Curves

**Figure 7:**
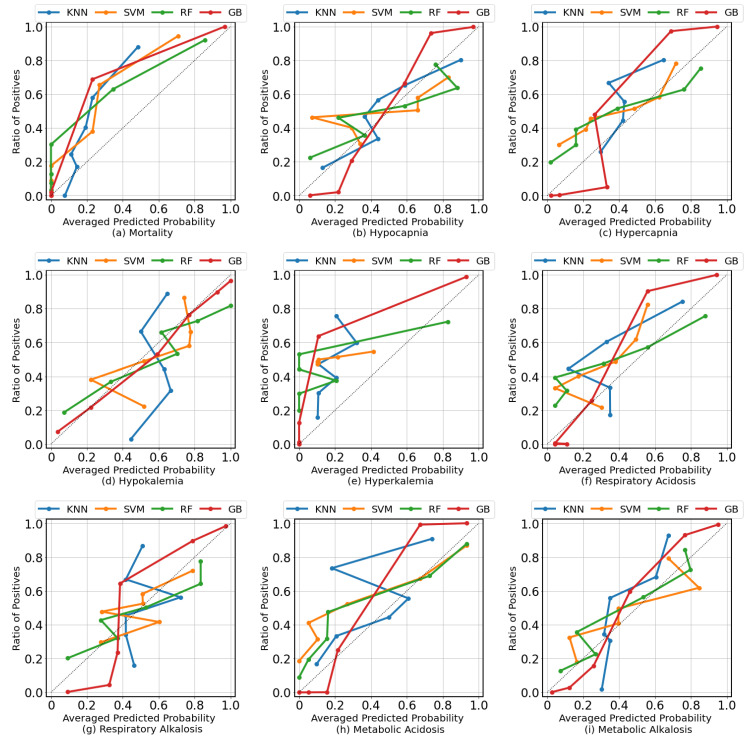
Calibration curves demonstrate the degree of calibration of each classifier for every clinical condition in the test set. Most of the calibration curves indicate that the classifiers are well-calibrated.

Figure 7 shows calibration curves that plot the mean predicted probability against the frequency of the event on the test set. A perfect model would have a calibration curve that is a straight line with a slope of one and an intercept of zero. If the curve is above the straight line, it means the model is overconfident, while a curve below the line means the model is underconfident. Calibration curves can be used to evaluate how well a model is able to predict events within different probability ranges. Compared with other algorithms, the GB models were better calibrated. The GB model of hypokalemia appears to be well-calibrated. The GB models seem to provide a risk score in the range between 0–1 better than other algorithms.

### 4.3. Feature Importance

Overall, the feature importance results in Figure 6 provide insight into the factors that the model is considering important when making predictions, which can help to increase understanding of the model’s decision-making process and inform further development or refinement of the model. Our results of feature importance indicate that the model had a strong understanding of the context surrounding the clinical conditions it was predicting. For mortality, the top features identified by the model (such as consciousness, SBP, SpO${}_{2}$, and RR) are included in commonly used ICU bedside scoring indexes, such as APACHE [10] and MEWS [9]. The models for predicting mortality were effective due, in part, to the inclusion of patient consciousness as input features. For hypocapnia and hypercapnia, pCO${}_{2}$ and TCO${}_{2}$, which are direct predictors of hypocapnia and hypercapnia, came up on top of the lists of feature importance. For hypokalemia and hyperkalemia, both types of potassium measurements from both chemistry labs and arterial blood gases were the most important variables. For acid–base imbalances, the variables indicating acid–base balance, electrolytes, and gasses in blood (such as SBE, ABE, pCO${}_{2}$, TCO${}_{2}$, cHCO${}_{3}$, K${}^{+}$, and Na${}^{+}$) are strong predictors. It is likely that the model, although not specifically designed to process temporal data, learned the temporal dynamics of the changes as well as the relationship between the different predictors to make predictions about the occurrence of these clinical conditions.

**Figure 8:**
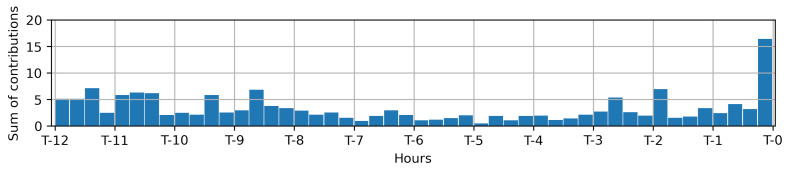
Temporal importance was calculated by averaging across all clinical variables and all clinical conditions in the test set. This measure reflects the degree of influence of each time point on the prediction. We observed that greater weight was given to the most recent and earlier measurements. This may indicate that the algorithms are able to capture changes in clinical variables over time.

In Figure 8, the temporal importance of each clinical variable was averaged across all clinical conditions in the test set. Hence, the model’s predictions were based on multiple time points, with the most recent and earlier measurements being weighted more heavily. This means that the model takes into account the temporal dynamics of the data when making predictions.

### 4.4. Feature Explanations

**Figure 9:**
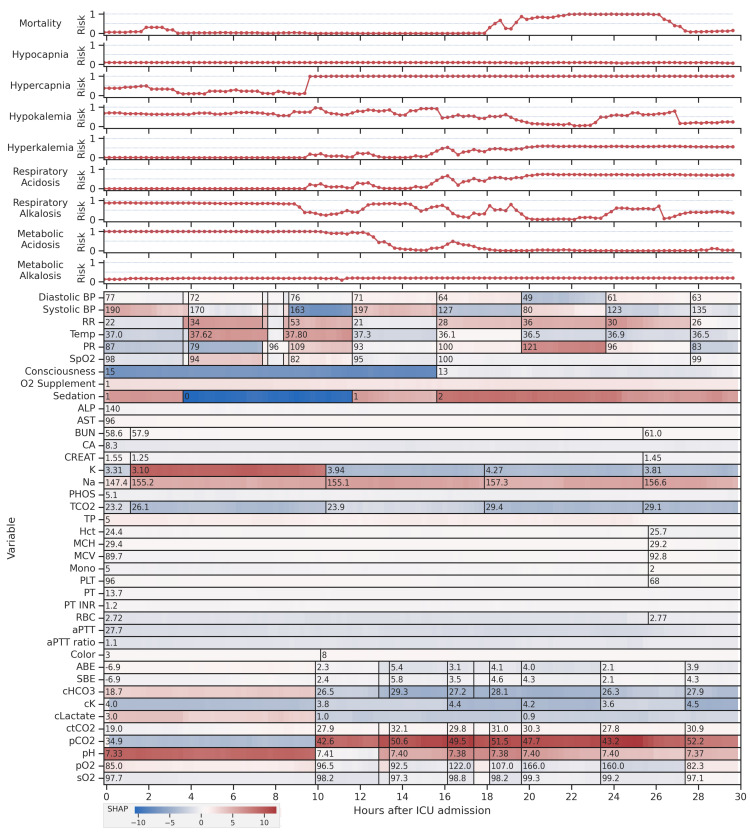
Feature visualization of one sample patient in the test set with model outputs provides valuable insights into how the models are making predictions based on the clinical data. By transforming the raw data into time series with a fixed 15 min interval and fill forward imputation, the models can analyze patterns over time and predict the patient’s score for the clinical condition. The models can also flag variables outside their normal range, indicating areas requiring attention from clinicians. It is worth noting that only the top 40 most important clinical variables are shown, and some measurements are skipped due to space limitations. Overall, the feature visualization with model outputs is a powerful tool for understanding how the models are making predictions and identifying areas where clinicians can take action to improve patient outcomes.

The explainability of models is critical in healthcare because healthcare providers are responsible for the actions taken. It is important that the decisions made by machine learning models are understandable to the user. Figure 9 presents a visualization of a sample patient in the test set over a period of 30 h, as well as the model outputs and the visualization of the contributions of each feature at each time step to the output of the model. The top 40 important clinical variables were selected for this visualization. The contributions were calculated using SHAP values from the SHAP framework [38], which were summed over different time steps of the same values and again summed over different clinical conditions. Some clinical variables, i.e., vital signs and arterial blood gases, were measured more frequent than the others. The models can identify variables that are outside of their normal ranges, e.g., the periods with low blood pressure and high respiratory rate, that may need attention of medical doctors.

### 4.5. Limitations

There are several limitations to this study. First, the dataset used was relatively small compared to larger datasets, such as MIMIC [39] and eICU [40], but the results do demonstrate that it is possible to develop algorithms using data from a single local institution. Second, our study defined acid–base disturbances using the simple thresholding technique, not from the point of view of the carbonic-acid–bicarbonate buffer system. Future studies may take into account the dynamics of the physiological regulation and buffer system. Third, the study did not consider other factors, such as medications or diagnoses made by physicians, which may have an impact on the results. The reason is that these data are too fine-grained, and it might be difficult for machine learning algorithms to learn from them effectively with a small dataset. If we take these factors into account, we could study the impact of chronic conditions on critically ill patients, which in turn can improve the performance of the algorithms. Expanding the dataset to include data from other ICUs within the same institution could help address this limitation. Fourth, balancing a dataset using methods, such as SMOTE (Synthetic Minority Over-sampling Technique) [41], can improve the performance of a machine learning model in minority class prediction by creating synthetic samples by interpolating between existing minority class samples and then increasing the number of minority class samples in the training dataset. Fifth, the study did not use more complex modeling techniques, such as recurrent neural networks or transformers, which have built-in temporal dynamics functionality, due to the desire to use smaller computational units and maintain interpretability of features. Additionally, marginal performance improvements of these complex modeling techniques were observed in other studies compared to ensemble tree algorithms. Finally, the study was conducted using data from a single institution, and the results may not be generalizable to other hospitals or healthcare settings.

### 4.6. Future Work

Future work involves examining the generalizability of the algorithms across different datasets, such as MIMIC and eICU, or with different cohorts. This can provide insight into how well the model would perform on a broader population. To achieve this, it is important to ensure that the variables used in the algorithm are also present in the target dataset. As suggested by the study by Desautels et al. [42], the performance and robustness of the algorithm can be improved through the use of transfer learning on a target dataset, such that knowledge learned from one dataset can be applied to improve the performance on a different but related dataset. Next, the performance of the model could potentially be improved when a model is trained with data from bedside monitoring systems, such as continuous vital signs. This is because bedside monitoring data provide a more detailed and granular view of a patient’s physiology and disease progression over time. With this type of data, the model is able to detect subtle changes in a patient’s condition that may not be apparent with less frequent measurements, such as those taken during routine care. The use of data from bedside monitoring systems would also allow the model to account for the dynamic nature of critical illness, which is characterized by rapid physiologic changes. Finally, it would be interesting to investigate whether the factors from bedside monitoring tools, such as APACHE II or SAPS, which are currently utilized in the clinic, can be used to predict acid–base and potassium imbalances. If these factors are found to be useful, they could be incorporated into clinical decision-making tools and treatment protocols without requiring additional data to be collected.

## 5. Conclusions

Acid–base disorders occur when there is an imbalance in the normal pH of the body. This can be caused by problems with kidney or respiratory function or by an excess of acids or bases that the body cannot properly eliminate. Acid–base disorders can also affect potassium levels in the body by altering the transport of potassium. It is important to monitor and regulate both acid–base balance and potassium levels to maintain proper physiological and cellular function of the body. This study used machine learning to predict acid–base and potassium imbalances in intensive care patients, which could be useful in controlling or regulating the balance and thus beneficial to the management of the underlying disease. We were interested in nine clinical conditions related to the acid–base and potassium imbalances: mortality, hypocapnia, hypercapnia, hypokalemia, hyperkalemia, metabolic acidosis, metabolic alkalosis, respiratory acidosis, and respiratory alkalosis. The study used an institutional dataset of 1089 patients with 87 clinical variables, including vital signs, patient appearance, and laboratory measurements. The results showed that GB generally had the best performance, with AUROCs ranging from 0.6767 to 0.9822 and AUPRCs ranging from 0.5945 to 0.9455 for the different clinical conditions and different prediction windows. The highest performances were seen in the prediction of mortality and hypokalemia, and the predictions for mortality, hypokalemia, and metabolic alkalosis remained relatively robust even when the prediction window was increased, indicating the potential for early prediction. We used the SHAP framework to make the decision-making process of our machine learning models interpretable and transparent. The results were promising and could be useful for clinicians to gain insights into the underlying clinical condition.

## Figures and Tables

**Table 1:** Dataset characteristics.

Characteristics	
Number of patients	1089
Number of admissions	1137
Age ${}^{1}$	63.8 (18.1)
Gender	
Male ${}^{2}$	602 (55.3%)
Female ${}^{2}$	487 (44.7%)
Length of hospital stay ${}^{1}$	27.2 (27.5)
Length of ICU stay ${}^{1}$	7.0 (8.0)

^1^ Values in Mean (S.D.); ^2^ Values in N (%).

**Table 2:** Criteria and statistics of clinical conditions.

Clinical Condition	Criteria	Admissions with Condition
Mortality	Death during ICU stay	213 (18.7%)
Hypocapnia	pCO_2_ < 35 mmHg	360 (31.7%)
Hypercapnia	pCO_2_ > 45 mmHg	296 (26.0%)
Hypokalemia	K^+^ < 3.5 mmol/L	598 (52.6%)
Hyperkalemia	K^+^ > 5.5 mmol/L	83 (7.3%)
Metabolic Acidosis	pH < 7.35, and cHCO_3_^−^ < 22 mmol/L	202 (17.8%)
Metabolic Alkalosis	pH > 7.45, and cHCO_3_^−^ > 26 mmol/L	430 (37.8%)
Respiratory Acidosis	pH < 7.35, and pCO_2_ > 45 mmHg	258 (22.7%)
Respiratory Alkalosis	pH > 7.45, and pCO_2_ < 35 mmHg	397 (34.9%)

**Table 3:** Performance for the next-hour prediction on each clinical condition on the test set.

Clinical Condition	N${}_{1}$/N${}_{0}$	Algorithm	Precision	Sensitivity	Specificity	F1 Score	AUROC	AUPRC
**Mortality**	213/	K Nearest Neighbours	0.3333	0.5625	0.7410	0.4186	0.7156	0.4347
	924	Support Vector Machine	0.4746	0.8750	0.7770	0.6154	0.9069	0.6796
		Random Forests	0.6154	**1.000**	0.8561	0.7619	0.9643	0.8378
		Gradient Boosting	**0.6809**	**1.000**	**0.8921**	**0.8101**	**0.9822**	**0.8557**
**Hypocapnia**	360/	K Nearest Neighbours	0.5484	0.6538	0.5625	0.5965	0.674	0.6353
	777	Support Vector Machine	0.6471	0.6346	0.7188	0.6408	0.6854	0.6282
		Random Forests	0.6429	0.6923	0.6875	0.6667	0.7263	0.615
		Gradient Boosting	**0.7115**	**0.7115**	**0.7656**	**0.7115**	**0.7524**	**0.6442**
**Hypercapnia**	296/	K Nearest Neighbours	0.381	0.4444	0.6422	0.4103	0.5817	0.4107
	841	Support Vector Machine	0.5063	0.7407	0.6422	0.6015	0.7397	0.5382
		Random Forests	**0.6056**	**0.7963**	**0.7431**	**0.6880**	**0.8255**	**0.7036**
		Gradient Boosting	0.5909	0.7222	0.7523	0.6500	0.8228	0.6731
**Hypokalemia**	598/	K Nearest Neighbours	0.6067	0.5684	0.4697	0.5870	0.5596	0.6779
	539	Support Vector Machine	0.75	0.7263	0.6515	0.738	0.6753	0.7359
		Random Forests	0.8061	0.8316	0.7121	0.8187	0.8593	0.8971
		Gradient Boosting	**0.8646**	**0.8737**	**0.8030**	**0.8691**	**0.9191**	**0.9455**
**Hyperkalemia**	83/	K Nearest Neighbours	0.0909	0.6000	0.6273	0.1579	0.5981	0.0714
	1054	Support Vector Machine	0.0921	0.7000	0.5714	0.1628	0.6441	0.0981
		Random Forests	0.1600	0.8000	0.7391	0.2667	0.854	0.3053
		Gradient Boosting	**0.2083**	**1.0000**	**0.7640**	**0.3448**	**0.9565**	**0.6497**
**Respiratory Acidosis**	202/	K Nearest Neighbours	0.2545	0.4375	0.6963	0.3218	0.5422	0.2938
	935	Support Vector Machine	0.3182	0.6562	0.6667	0.4286	0.6484	0.2797
		Random Forests	**0.4237**	**0.7812**	**0.7481**	**0.5495**	0.7995	0.5324
		Gradient Boosting	0.4068	0.7500	0.7407	0.5275	**0.8125**	**0.5945**
**Respiratory Alkalosis**	430/	K Nearest Neighbours	0.4405	0.5873	0.5204	0.5034	0.5234	0.4175
	707	Support Vector Machine	0.4815	0.6190	0.5714	0.5417	0.6260	0.5335
		Random Forests	**0.6269**	0.6667	**0.7449**	0.6462	0.7517	0.6265
		Gradient Boosting	0.6081	**0.7143**	0.7041	**0.6569**	**0.7685**	**0.6924**
**Metabolic Acidosis**	258/	K Nearest Neighbours	0.3492	0.5641	0.6639	0.4314	0.6783	0.4081
	879	Support Vector Machine	0.4648	0.8462	0.6885	0.6000	0.8600	0.5921
		Random Forests	**0.5385**	**0.8974**	**0.7541**	**0.6731**	**0.8699**	0.6870
		Gradient Boosting	0.5333	0.8205	0.7705	0.6465	0.8682	**0.7150**
**Metabolic Alkalosis**	397/	K Nearest Neighbours	0.4583	0.5690	0.6176	0.5077	0.6228	0.5258
	740	Support Vector Machine	0.5846	0.6552	0.7353	0.6179	0.7329	0.6153
		Random Forests	0.5946	0.7586	0.7059	0.6667	0.7694	0.6143
		Gradient Boosting	**0.6389**	**0.7931**	**0.7451**	**0.7077**	**0.8284**	**0.6719**

Bold texts highlight the highest scores in each metric for each clinical condition. N_1_ represents the number of samples with positive outcomes. N_0_ represents the number of samples with positive outcomes. (Total = 1137).

**Table 4:** Predictive performance of the GB classifier at different prediction windows (T${}_{P}$ = {1,2,4,8}).

Clinical Condition	N${}_{1}$/N${}_{0}$	Before Onset	Precision	Sensitivity	Specificity	F1 Score	AUROC	AUPRC
**Mortality**	213/	1 h	0.6809	1.000	0.8921	0.8101	0.9822	0.8557
	924	2 h	0.6078	0.9688	0.8561	0.7470	0.9584	0.8148
		4 h	0.5769	0.9375	0.8417	0.7143	0.9670	0.8820
		8 h	0.5400	0.8438	0.8345	0.6585	0.9490	0.8462
**Hypocapnia**	360/	1 h	0.7115	0.7115	0.7656	0.7115	0.7524	0.6442
	777	2 h	0.6491	0.7255	0.6923	0.6852	0.7629	0.7207
		4 h	0.6034	0.7292	0.6515	0.6604	0.7105	0.5997
		8 h	0.4808	0.6757	0.6143	0.5618	0.7232	0.6099
**Hypercapnia**	296/	1 h	0.5909	0.7222	0.7523	0.6500	0.8228	0.6731
	841	2 h	0.5217	0.6792	0.6972	0.5902	0.7881	0.6081
		4 h	0.5600	0.7925	0.6972	0.6562	0.7880	0.6496
		8 h	0.4857	0.6667	0.6727	0.5620	0.7660	0.6142
**Hypokalemia**	598/	1 h	0.8646	0.8737	0.8030	0.8691	0.9191	0.9455
	539	2 h	0.8191	0.828	0.7500	0.8235	0.8885	0.9127
		4 h	0.7895	0.8427	0.7059	0.8152	0.8516	0.8866
		8 h	0.7907	0.8000	0.7429	0.7953	0.8309	0.8475
**Hyperkalemia**	83/	1 h	0.2083	1.0000	0.7640	0.3448	0.9565	0.6497
	1054	2 h	0.1579	0.9000	0.7019	0.2687	0.8981	0.2761
		4 h	0.1636	0.9000	0.7143	0.2769	0.8043	0.1573
		8 h	0.1667	0.9000	0.7205	0.2812	0.7882	0.1628
**Respiratory Acidosis**	202/	1 h	0.4068	0.7500	0.7407	0.5275	0.8125	0.5945
	935	2 h	0.3382	0.7419	0.6667	0.4646	0.7890	0.5339
		4 h	0.3607	0.7097	0.7111	0.4783	0.7728	0.4539
		8 h	0.3333	0.6129	0.7185	0.4318	0.7226	0.3975
**Respiratory Alkalosis**	430/	1 h	0.6081	0.7143	0.7041	0.6569	0.7685	0.6924
	707	2 h	0.6618	0.7143	0.7653	0.6870	0.7577	0.6250
		4 h	0.5733	0.7167	0.6768	0.6370	0.7608	0.6250
		8 h	0.5082	0.5962	0.7030	0.5487	0.6767	0.5167
**Metabolic Acidosis**	258/	1 h	0.5333	0.8205	0.7705	0.6465	0.8682	0.7150
	879	2 h	0.5000	0.8378	0.7480	0.6263	0.8548	0.6490
		4 h	0.4918	0.8108	0.7459	0.6122	0.8702	0.6686
		8 h	0.4375	0.8485	0.7073	0.5773	0.8559	0.6923
**Metabolic Alkalosis**	397/	1 h	0.6389	0.7931	0.7451	0.7077	0.8284	0.6719
	740	2 h	0.5970	0.7143	0.7404	0.6504	0.8110	0.6571
		4 h	0.5455	0.7636	0.6602	0.6364	0.8104	0.6775
		8 h	0.5513	0.7818	0.6602	0.6466	0.8095	0.6799

N_1_ represents the number of samples with positive outcomes. N_0_ represents the number of samples with positive outcomes. (Total = 1137).

## Data Availability

The de-identified clinical data presented in this study are available on request from the corresponding author, subject to approval by the Office of Human Research Ethic Committee. The data are not publicly available due to institutional policies.

## Supplementary Materials

The following supporting information can be downloaded at: https://www.mdpi.com/article/10.3390/diagnostics13061171/s1, Table S1: Summary of the functions in the scikit-learn library and the hyperparameters that are optimized during the training of an algorithm; Figure S1: Visualization of F1 score, AUROC, and AUPRC for each algorithm and for each clinical condition; Figure S2: Visualization of F1 score, AUROC, and AUPRC for each early prediction window and for each clinical condition; Figure S3: PR curves compare different algorithms for each clinical condition; Figure S4: PR curves compare different early prediction windows for each clinical condition; Figure S5: Confusion matrices of GB classifiers on the test set for a one hour early prediction window for each clinical condition.

## Abbreviations

The following abbreviations are used in this manuscript:

ABE	Actual Base Excess
ALB	Albumin
ALP	Alkaline Phosphatase
ALT	Alanine Transaminase
APACHE	Acute Physiology And Chronic Health Evaluation
aPTT	Activated Partial Thromboplastin Time
AST	Aspartate Transaminase
AUPRC	Area Under The Precision Recall Curve
AUROC	Area Under The Receiver Operating Characteristic Curve
BUN	Blood Urea Nitrogen
CA	Calcium
cCa_2_^+^	Calcium Concentration
cCl^−^	Chloride Concentration
cHCO_3_^−^	Bicarbonate Concentration
cK^+^	Potassium Concentration
CKD	Chronic Kidney Disease
Cl^−^	Chloride
cLac	Lactate Concentration
cNa^+^	Sodium Concentration
CO${}_{2}$	Carbon Dioxide
CREAT	Creatinine
ctCO_2_	Total Concentration Of Carbon Dioxide
DBP	Diastolic Blood Pressure
EHR	Electronic Health Record
EWS	Early Warning Score
GB	Gradient Boosting
GLOB	Globulin
ICU	Intensive Dare Unit
K^+^	Potassium
KNN	*K* Nearest Neighbours
LSTM	Long Short-Term Memory
MCH	Mean Corpuscular Hemoglobin
MCHC	Mean Corpuscular Hemoglobin Concentration
MCV	Mean Corpuscular Volume
MEWS	Modified Early Warning Score
MICU	Medical Intensive Care Unit
Mono	Monocyte
Na^+^	Sodium
NEWS	National Early Warning Score
pCO_2_	Partial Pressure Of Carbon Dioxide
PHOS	Phosphate
PLT	Platelet
pO_2_	Partial Pressure Of Oxygen
PR	Pulse Rate
PT	Prothrombin Time
RBC	Red Blood Cell
RF	Random Forest
ROC	Receiver Operating Characteristic
RR	Respiratory Rate
SAPS	Simplified Acute Physiology Score
SBE	Standard Base Excess
SBP	Systolic Blood Pressure
SHAP	Shapley Additive Explanations
SIRS	Systemic Inflammatory Response Syndrome
sO_2_	Oxygen Saturation
SpO_2_	Oxygen Saturation
SQL	Structured Query Language
SVM	Support Vector Machine
TCO_2_	Total Carbon Dioxide
TP	Total Protein

## References

[1] Adhikari N.K., Fowler R.A., Bhagwanjee S., Rubenfeld G.D. (2010). Critical care and the global burden of critical illness in adults. Lancet.

[2] Bhagavan N., Ha C.E. (2015). Water, Electrolytes, and Acid–Base Balance. Essentials of Medical Biochemistry.

[3] Quinteros L.M., Roque J.B., Kaufman D., Raventós A.A. (2019). Importance of carbon dioxide in the critical patient: Implications at the cellular and clinical levels. Med. Intensiv. (Engl. Ed.).

[4] Forsal I., Bodelsson M., Wieslander A., Nilsson A., Pouchoulin D., Broman M. (2022). Analysis of acid–base disorders in an ICU cohort using a computer script. Intensive Care Med. Exp..

[5] Hamm L.L., Hering-Smith K.S., Nakhoul N.L. (2013). Acid-Base and Potassium Homeostasis. Semin. Nephrol..

[6] Kazda A., Jabor A., Zámečník M., Mašek K. (1989). Monitoring Acid-Base and Electrolyte Disturbances in Intensive Care. Advances in Clinical Chemistry.

[7] Adrogué H.J., Madias N.E. (1981). Changes in plasma potassium concentration during acute acid–base disturbances. Am. J. Med..

[8] Charlton P.H., Pimentel M., Lokhandwala S. (2016). Data Fusion Techniques for Early Warning of Clinical Deterioration. Secondary Analysis of Electronic Health Records.

[9] Subbe C. (2001). Validation of a modified Early Warning Score in medical admissions. QJM.

[10] Knaus W.A., Draper E.A., Wagner D.P., Zimmerman J.E. (1985). APACHE II: A Severity of Disease Classification System. Crit. Care Med..

[11] Moreno R.P., Metnitz P.G.H., Almeida E., Jordan B., Bauer P., Campos R.A., Iapichino G., Edbrooke D., Capuzzo M., Le Gall J.-R. (2005). SAPS 3—From evaluation of the patient to evaluation of the intensive care unit. Part 2: Development of a prognostic model for hospital mortality at ICU admission. Intensive Care Med..

[12] Jones M. (2012). NEWSDIG: The National Early Warning Score Development and Implementation Group. Clin. Med..

[13] Cosgriff C.V., Celi L.A., Stone D.J. (2019). Critical Care, Critical Data. Biomed. Eng. Comput. Biol..

[14] Johnson A.E.W., Ghassemi M.M., Nemati S., Niehaus K.E., Clifton D., Clifford G.D. (2016). Machine Learning and Decision Support in Critical Care. Proc. IEEE.

[15] Kam H.J., Kim H.Y. (2017). Learning representations for the early detection of sepsis with deep neural networks. Comput. Biol. Med..

[16] Nemati S., Holder A., Razmi F., Stanley M.D., Clifford G.D., Buchman T.G. (2018). An Interpretable Machine Learning Model for Accurate Prediction of Sepsis in the ICU. Crit. Care Med..

[17] Zhang D., Yin C., Hunold K.M., Jiang X., Caterino J.M., Zhang P. (2021). An interpretable deep-learning model for early prediction of sepsis in the emergency department. Patterns.

[18] Kwon J., Lee Y., Lee Y., Lee S., Park J. (2018). An Algorithm Based on Deep Learning for Predicting In-Hospital Cardiac Arrest. J. Am. Heart Assoc..

[19] Tomašev N., Glorot X., Rae J.W., Zielinski M., Askham H., Saraiva A., Mottram A., Meyer C., Ravuri S., Protsyuk I. (2019). A clinically applicable approach to continuous prediction of future acute kidney injury. Nature.

[20] Wanyan T., Honarvar H., Jaladanki S.K., Zang C., Naik N., Somani S., Freitas J.K.D., Paranjpe I., Vaid A., Zhang J. (2021). Contrastive learning improves critical event prediction in COVID-19 patients. Patterns.

[21] Lee J.M., Hauskrecht M. (2021). Modeling multivariate clinical event time-series with recurrent temporal mechanisms. Artif. Intell. Med..

[22] Kaji D.A., Zech J.R., Kim J.S., Cho S.K., Dangayach N.S., Costa A.B., Oermann E.K. (2019). An attention based deep learning model of clinical events in the intensive care unit. PLoS ONE.

[23] Na Pattalung T., Chaichulee S. (2021). Comparison of machine learning algorithms for mortality prediction in intensive care patients on multi-center critical care databases. IOP Conf. Ser. Mater. Sci. Eng..

[24] Na Pattalung T., Ingviya T., Chaichulee S. (2021). Feature Explanations in Recurrent Neural Networks for Predicting Risk of Mortality in Intensive Care Patients. J. Pers. Med..

[25] Chen H., Lundberg S.M., Erion G., Kim J.H., Lee S.I. (2021). Forecasting adverse surgical events using self-supervised transfer learning for physiological signals. NPJ Digit. Med..

[26] Fan Y., Ye T., Huang T., Xiao H. (2022). Machine learning-based construction of a clinical prediction model for hypercapnia during one-lung ventilation for lung surgery. Res. Sq..

[27] Zhou Z., Huang C., Fu P., Huang H., Zhang Q., Wu X., Yu Q., Sun Y. (2022). Prediction of in-hospital hypokalemia using machine learning and first hospitalization day records in patients with traumatic brain injury. CNS Neurosci. Ther..

[28] Kwak G.H., Chen C., Ling L., Ghosh E., Celi L.A., Hui P. (2021). Predicting Hyperkalemia in the ICU and Evaluation of Generalizability and Interpretability. arXiv.

[29] Cherif A., Maheshwari V., Fuertinger D., Schappacher-Tilp G., Preciado P., Bushinsky D., Thijssen S., Kotanko P. (2020). A mathematical model of the four cardinal acid–base disorders. Math. Biosci. Eng..

[30] Laserna E., Sibila O., Aguilar P.R., Mortensen E.M., Anzueto A., Blanquer J.M., Sanz F., Rello J., Marcos P.J., Velez M.I. (2012). Hypocapnia and Hypercapnia Are Predictors for ICU Admission and Mortality in Hospitalized Patients With Community-Acquired Pneumonia. Chest.

[31] Soar J., Perkins G.D., Abbas G., Alfonzo A., Barelli A., Bierens J.J., Brugger H., Deakin C.D., Dunning J., Georgiou M. (2010). European Resuscitation Council Guidelines for Resuscitation 2010 Section 8. Cardiac arrest in special circumstances: Electrolyte abnormalities, poisoning, drowning, accidental hypothermia, hyperthermia, asthma, anaphylaxis, cardiac surgery, trauma, pregnancy, electrocution. Resuscitation.

[32] Berend K., de Vries A.P., Gans R.O. (2014). Physiological Approach to Assessment of Acid–Base Disturbances. N. Engl. J. Med..

[33] Constable P.D. (2000). Clinical Assessment of Acid-Base Status: Comparison of the Henderson-Hasselbalch and Strong Ion Approaches. Vet. Clin. Pathol..

[34] Rawat D., Modi P., Sharma S. (2022). Hypercapnea.

[35] Gennari F.J. (1998). Hypokalemia. N. Engl. J. Med..

[36] GALLA J.H. (2000). Metabolic Alkalosis. J. Am. Soc. Nephrol..

[37] Plant P.K. (2000). One year period prevalence study of respiratory acidosis in acute exacerbations of COPD: Implications for the provision of non-invasive ventilation and oxygen administration. Thorax.

[38] Lundberg S.M., Erion G., Chen H., DeGrave A., Prutkin J.M., Nair B., Katz R., Himmelfarb J., Bansal N., Lee S.I. (2020). From local explanations to global understanding with explainable AI for trees. Nat. Mach. Intell..

[39] Johnson A., Bulgarelli L., Pollard T., Horng S., Celi L.A., Mark R. (2022). MIMIC-IV. Phys. Net..

[40] Pollard T.J., Johnson A.E.W., Raffa J.D., Celi L.A., Mark R.G., Badawi O. (2018). The eICU Collaborative Research Database, a freely available multi-center database for critical care research. Sci. Data.

[41] Cihan P., Ozger Z.B. (2022). A new approach for determining SARS-CoV-2 epitopes using machine learning-based in silico methods. Comput. Biol. Chem..

[42] Desautels T., Calvert J., Hoffman J., Mao Q., Jay M., Fletcher G., Barton C., Chettipally U., Kerem Y., Das R. (2017). Using Transfer Learning for Improved Mortality Prediction in a Data-Scarce Hospital Setting. Biomed. Inform. Insights.

