# Supplementary material for: Prediction of Acid-Base and Potassium Imbalances in Intensive Care Patients Using Machine Learning Techniques
Source: Diagnostics (Basel). 2023 Mar 18;13(6):1171. doi: 10.3390/diagnostics13061171 (PMC10047445; doi:10.3390/diagnostics13061171)
Supplement: Supplementary file 1 [file diagnostics-13-01171-s001.zip › diagnostics-2235807-supplementary.pdf]

Supplementary Materials

Prediction of Acid–Base and Potassium Imbalances in Intensive Care Patients using Machine Learning Techniques

Ratchakit Phetrittikun, Kerdkiat Suvirat, Kanakorn Horsiritham,
Thammasin Ingviya, and Sitthichok Chaichulee

Table S1: Summary of the functions in the scikit-learn library and the hyperparameters that are optimized during the training of an algorithm.

**Algorithm**	**Function in `scikit-learn`**	**Optimising hyperparameter**
K Nearest Neighbours	`KNeighborsClassifier`	Number of neighbors : {3, 5, 7, 9}<br>Weight function: {'uniform','distance'}
Support Vector Machine	`SVC`	Kernel function: {'radial basis function', 'polynomial'}<br>C parameters: {1, 10, 100, 1000}
Random Forests	`RandomForestClassifier`	Number of trees: {200, 400, 600}<br>Maximum depth of the tree: {4, 8, 12, 16}
Gradient Boosting	`GradientBoostingClassifier`	Number of trees: {100, 250, 400}<br>Maximum depth of the tree: {4, 8, 12}

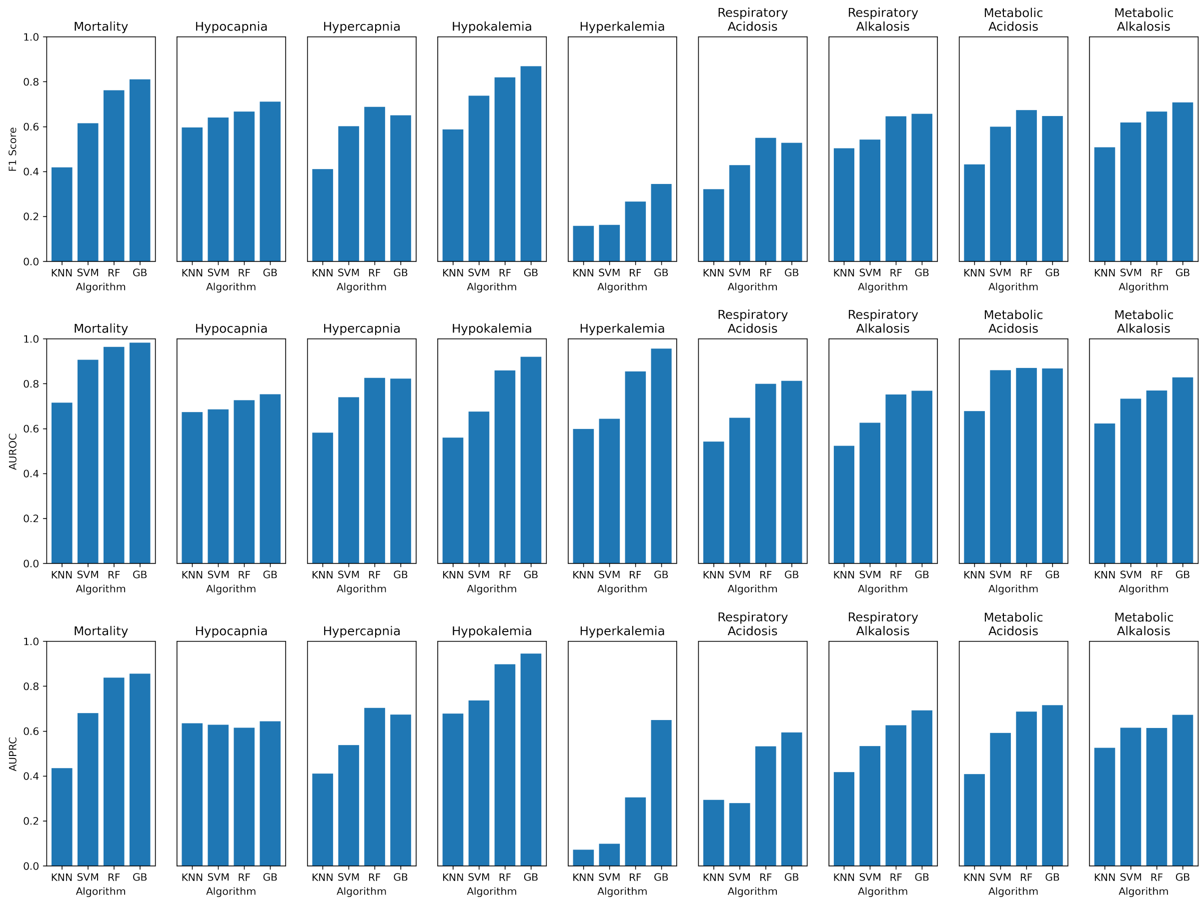

Figure S1: Visualization of F1 score, AUROC, and AUPRC for each algorithm and for each clinical condition. The data are taken from Table 3

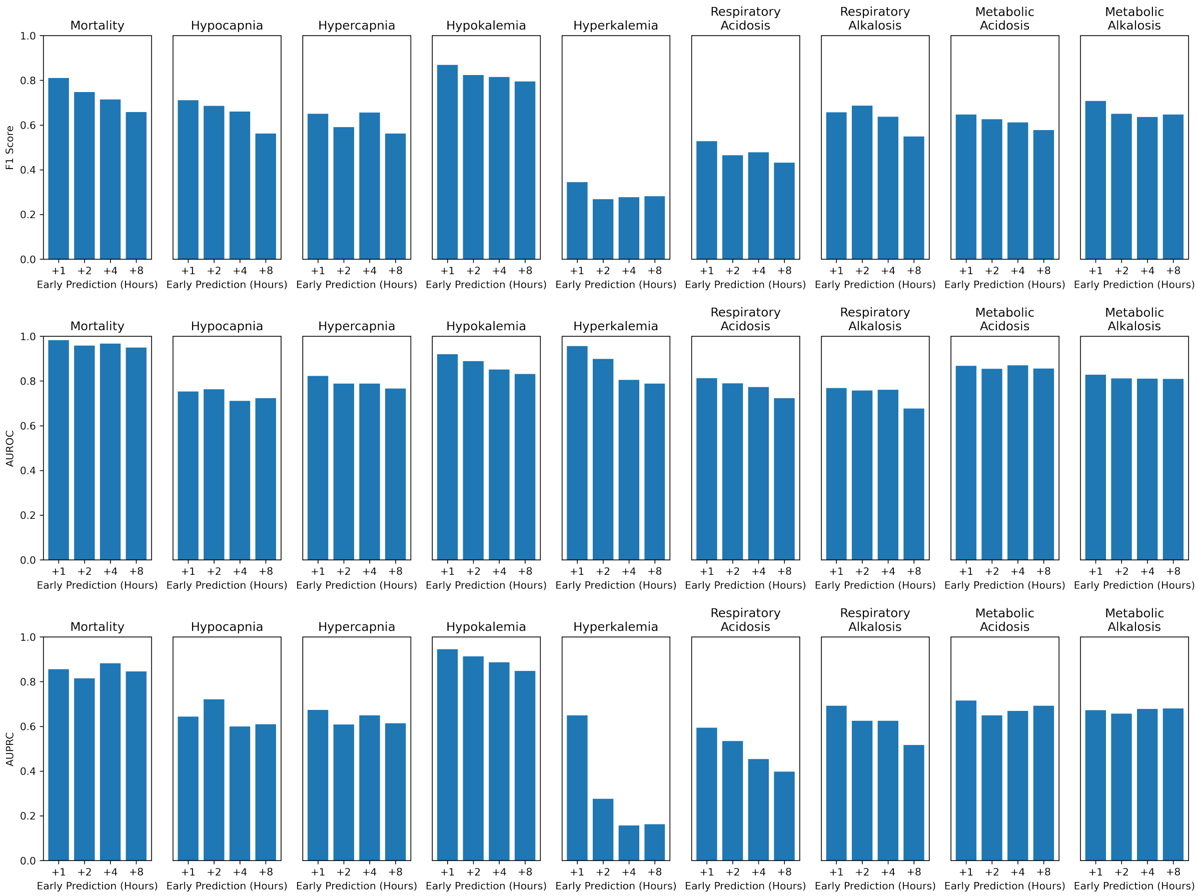

Figure S2: Visualization of F1 score, AUROC, and AUPRC for each early prediction window ({1, 2, 4, 8}) and for each clinical condition. The data are taken from Table 3

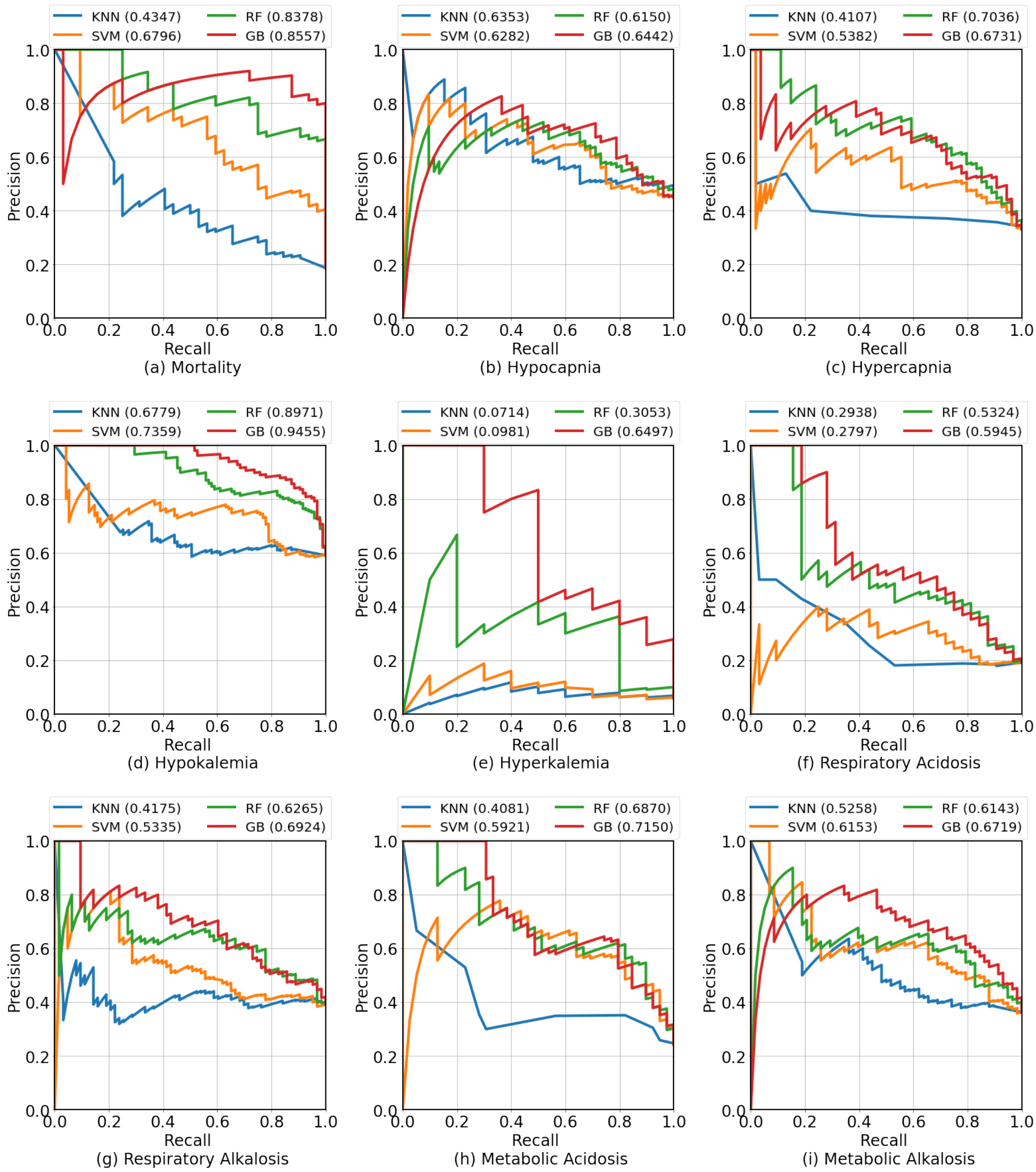

Figure S3: PR curves compare different algorithms for each clinical condition: (a) mortality, (b) hypocapnia, (c) hypercapnia, (d) hypokalemia, (e) hyperkalemia, (f) respiratory acidosis, (g) respiratory alkalosis, (h) metabolic acidosis, and (i) metabolic alkalosis.

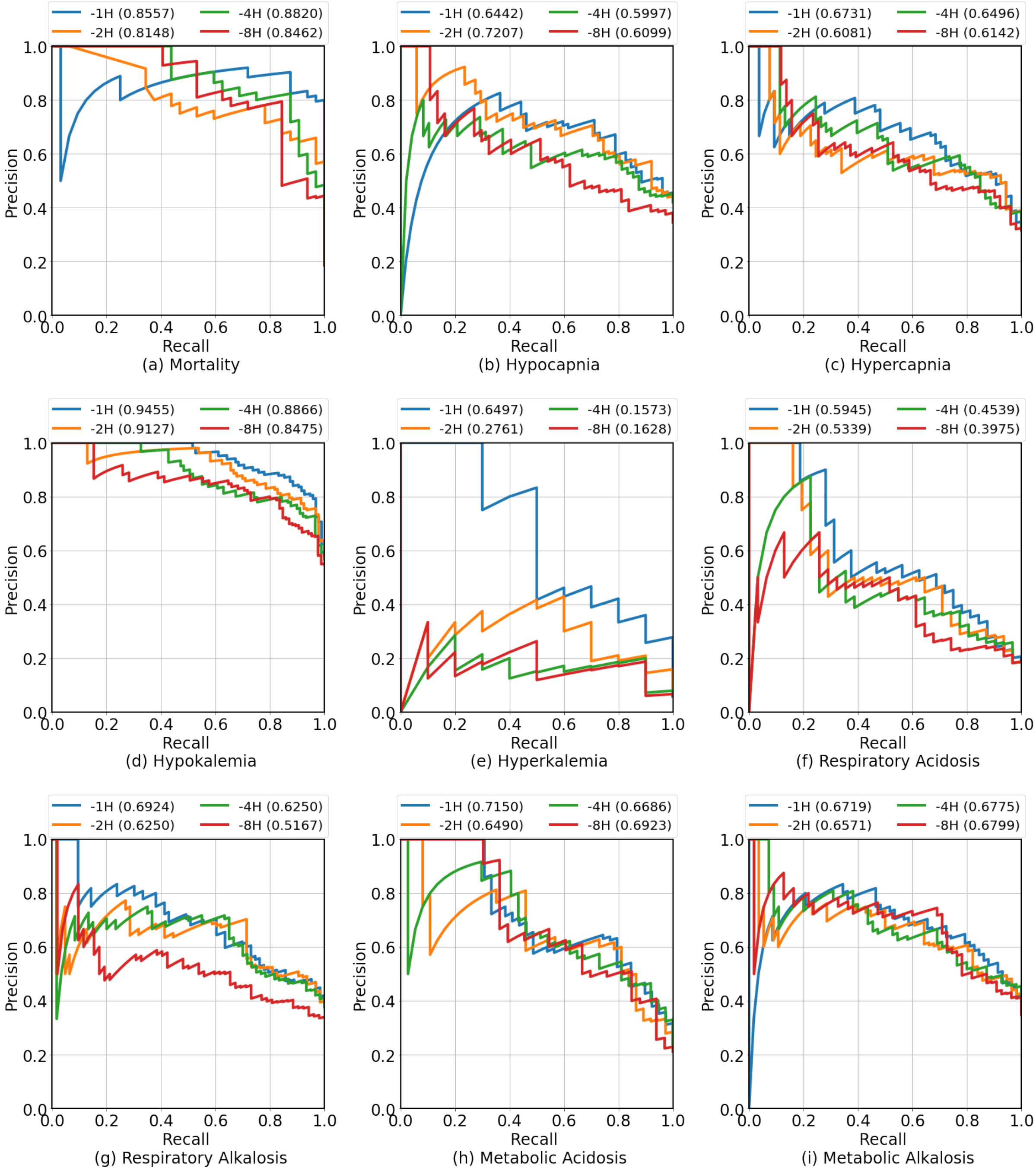

Figure S4: PR curves compare different early prediction windows for each clinical condition: (a) mortality, (b) hypocapnia, (c) hypercapnia, (d) hypokalemia, (e) hyperkalemia, (f) respiratory acidosis, (g) respiratory alkalosis, (h) metabolic acidosis, and (i) metabolic alkalosis.

(a) Mortality

		Actual condition	
		Negative	Positive
Prediction	Negative	124	0
	Positive	15	32

(b) Hypocapnia

		Actual condition	
		Negative	Positive
Prediction	Negative	49	15
	Positive	15	37

(c) Hypercapnia

		Actual condition	
		Negative	Positive
Prediction	Negative	82	15
	Positive	27	39

(d) Hypokalemia

		Actual condition	
		Negative	Positive
Prediction	Negative	53	12
	Positive	13	83

(e) Hyperkalemia

		Actual condition	
		Negative	Positive
Prediction	Negative	123	0
	Positive	38	10

(f) Respiratory Acidosis

		Actual condition	
		Negative	Positive
Prediction	Negative	100	8
	Positive	35	24

(g) Respiratory Alkalosis

		Actual condition	
		Negative	Positive
Prediction	Negative	69	18
	Positive	29	45

(h) Metabolic Acidosis

		Actual condition	
		Negative	Positive
Prediction	Negative	94	7
	Positive	28	32

(i) Metabolic Alkalosis

		Actual condition	
		Negative	Positive
Prediction	Negative	76	12
	Positive	26	46

Figure S5: Confusion matrices of GB classifiers on the test set for a one hour early prediction window for each clinical condition: (a) mortality, (b) hypocapnia, (c) hypercapnia, (d) hypokalemia, (e) hyperkalemia, (f) respiratory acidosis, (g) respiratory alkalosis, (h) metabolic acidosis, and (i) metabolic alkalosis.
